# Role of Serum Procalcitonin in Predicting the 30-Day Postoperative Mortality Rate in the Elderly Population: A Systematic Review and Meta-Analysis

**DOI:** 10.7759/cureus.90869

**Published:** 2025-08-24

**Authors:** Khong Wee Lee, Khairina Khairuddin, Tarun Bhuvanagiri, Lauren Oliver

**Affiliations:** 1 Trauma and Orthopaedics, Ysbyty Gwynedd, Bangor, GBR; 2 Public Health, Birmingham City University, Bangor, GBR; 3 Orthogeriatrics, Ysbyty Gwynedd, Bangor, GBR

**Keywords:** biomarker kinetics, elderly surgical patients, mortality prediction, orthogeriatric care, postoperative infections, procalcitonin

## Abstract

Postoperative complications are a leading cause of morbidity and mortality among elderly patients, largely due to age-related physiological decline, increased comorbidities, and heightened susceptibility to infection. Procalcitonin (PCT), a peptide biomarker associated with systemic bacterial infection, has gained recognition for its diagnostic relevance in critical care and infectious disease settings. While PCT has shown promise in identifying sepsis and guiding antimicrobial therapy, its utility in surgical contexts, especially within geriatric populations, remains incompletely defined. This review aims to evaluate the predictive capacity of perioperative PCT measurements in estimating 30-day mortality and infection-related outcomes in elderly surgical patients, offering insight into its potential role in perioperative risk stratification and management. A systematic review was conducted using 11 studies involving patients aged 65 years and older who underwent cardiac, orthopaedic, thoracic, abdominal, or general surgery. Eligible studies were selected based on their evaluation of serum PCT levels measured perioperatively and the documented association with either 30-day postoperative mortality or infectious complications. Key parameters, such as study design, patient demographics, timing of PCT measurement, biomarker thresholds, and outcome definitions, were extracted. A narrative synthesis was performed, supplemented by pooled analysis of PCT kinetic trends to identify consistent patterns across clinical scenarios. Serial PCT measurements were consistently elevated in patients who developed postoperative infections, with values ranging from 0.69 to 1.16 ng/mL between days 3 and 8 post-surgery, compared to 0.03 to 0.18 ng/mL in non-infected controls (*p* < 0.001). Most infected patients showed peak levels within 48-72 hours postoperatively, while non-infected individuals exhibited transient elevations that resolved within the same period. Although four studies demonstrated significant associations between elevated PCT and early mortality, particularly when levels exceeded 0.8 ng/mL, the predictive value was inconsistent across other datasets. Mortality prediction improved when PCT was interpreted alongside clinical scoring systems such as multiple organ dysfunction syndrome (MODS) or systemic inflammatory response syndrome (SIRS), highlighting the benefit of multimodal assessment. PCT is a valuable biomarker for identifying postoperative infections in elderly surgical patients, particularly when measured in serial intervals and contextualised with clinical parameters. Its dynamic profile allows for early identification of infectious complications and supports timely intervention strategies. However, PCT’s role in mortality prediction is less consistent when used in isolation, showing improved reliability when integrated with systemic assessment tools. Standardised protocols and large-scale multicenter studies are needed to validate its prognostic application and optimise perioperative surveillance in high-risk geriatric cohorts.

## Introduction and background

With the global ageing population steadily increasing, the number of elderly patients undergoing surgical interventions has risen substantially. Estimates suggest that individuals aged 65 and older account for more than 30% of total surgical procedures worldwide annually, reflecting both the rising life expectancy and the growing demand for postoperative treatments [1]. While surgical advancements have significantly improved outcomes, elderly patients remain uniquely vulnerable to postoperative complications due to physiological decline, preexisting comorbidities, and impaired immune responses [2]. Based on data from the National Emergency Laparotomy Audit (NELA) in 2020, patients aged 65 years and older experienced significantly worse postoperative outcomes compared to their younger counterparts. Specifically, older adults had markedly higher 30-day (15.3% vs. 4.9%, P < 0.001) and 90-day mortality rates (20.4% vs. 7.2%, P < 0.001) and longer median hospital stays (15.2 vs. 11.3 days, P < 0.001) and were more frequently discharged to care-home accommodation (6.7% vs. 1.9%, P < 0.001) [2]. These findings underscore the heightened vulnerability of older patients undergoing emergency surgery and highlight the need for tailored perioperative strategies in this age group. Understanding and mitigating these risks is crucial for optimising perioperative care and improving survival rates in this population.

Perioperative inflammatory responses play a pivotal role in postoperative morbidity and mortality [3]. In elderly patients, these responses are often exacerbated by pre-existing systemic conditions such as cardiovascular disease, diabetes, and chronic inflammatory disorders, leading to heightened susceptibility to complications, including sepsis, organ dysfunction, and impaired wound healing. The dysregulation of inflammatory mediators can contribute to systemic inflammation, prolonging recovery and increasing mortality risk. Identifying reliable biomarkers that can effectively predict adverse outcomes is essential to enhancing patient management and guiding early interventions.

Procalcitonin (PCT) has emerged as a potential biomarker for systemic inflammation, with reasonable specificity in identifying infections, particularly bacterial sepsis [4]. As a pro-inflammatory peptide precursor of calcitonin, PCT levels rise in response to severe systemic infections and inflammatory insults. However, its prognostic value in perioperative settings, particularly in predicting postoperative mortality in elderly patients, remains inadequately explored. There remains a paucity of evidence elucidating the correlation between PCT levels and the 30-day mortality rate among elderly patients. Addressing this gap in knowledge could provide valuable insights into postoperative risk stratification, enabling clinicians to implement more targeted therapeutic strategies.

This systematic review and meta-analysis aim to examine the association between serum PCT levels and 30-day mortality following surgery in elderly patients. By synthesising existing literature and evaluating the predictive utility of PCT, this study seeks to contribute to evidence-based perioperative management strategies that could enhance surgical safety and patient survival in high-risk populations.

## Review

Materials and methods

This systematic review followed a predefined protocol informed by the guidance of the Centre for Reviews and Dissemination [5] and the Cochrane Handbook [6]. Reporting adhered to the standards outlined in the Preferred Reporting Items for Systematic reviews and Meta-Analyses (PRISMA) statement [7]. Studies were eligible for inclusion if they were published in English and specifically examined the relationship between serum PCT levels and 30-day postoperative mortality among patients aged 65 years or older. Only studies that assessed PCT in postoperative contexts were considered. Exclusion criteria included investigations involving contaminated or dirty surgical procedures, as well as studies that included patients who were critically ill at the time of presentation.

Databases including Cochrane, PsycINFO, MEDLINE, and PubMed were systematically searched using tailored keyword combinations. Examples of keywords incorporated in the strategy included “elderly”, “postoperative”, “procalcitonin”, and “mortality”. To minimise publication bias and ensure comprehensive coverage, specific Boolean-based search strings were constructed and primarily executed within PubMed/MEDLINE, Cochrane, and PsycINFO, as demonstrated in Table 1.

**Table 1:** Search steps

Search number	Search terms
Search 1 (P)	"elderly" OR "older patients" OR "geriatric"
Search 2 (P)	"postoperative" OR "post-surgery" OR "post-op"
Search 3 (I)	"pro calcitonin" OR "procalcitonin" OR "PCT"
Search 4 (O)	"death" OR "mortality" OR "survival"
Search 5	Search 1 AND Search 2 AND Search 3 AND Search 4

Screening of search results was conducted in two structured stages. Initially, titles and abstracts were assessed against predefined inclusion criteria. Studies meeting these criteria - or those deemed ambiguous - were advanced to the second phase. In this subsequent stage, full-text articles underwent detailed evaluation by three independent reviewers. Any discrepancies in judgment were resolved through discussion with the fourth reviewer and consensus among the reviewers. Selected articles underwent independent screening for inclusion criteria and writing quality, followed by source validation. A final list of eligible studies was compiled and subsequently assessed using the Mixed Methods Appraisal Tool [8]. Designed to evaluate methodological rigour across qualitative, quantitative, and mixed-methods designs, the MMAT is suitable for empirical studies but excludes non-empirical work, such as theoretical papers and literature reviews. Although not intended for designs like economic or diagnostic accuracy studies, the MMAT remains a widely endorsed tool for systematic mixed-methods reviews, refined through expert input and continuous user feedback.

Data extraction was conducted independently by two reviewers, with discrepancies resolved through discussion to ensure consensus. For each eligible study, key parameters were systematically captured, including study characteristics such as author names, publication year, design type, setting, and sample size. Population details were documented, encompassing age ranges, comorbid conditions, surgical categories, and postoperative status. Metrics specific to PCT were recorded, including the timing of measurement, baseline and follow-up levels, and threshold values used. Outcomes of interest included 30-day mortality rates, presence of surgical complications such as prosthetic joint infections, and hospital-acquired infections. In addition, diagnostic and prognostic performance indicators, such as sensitivity, specificity, predictive values, and clinical relevance for decision-making, were extracted to support synthesis and interpretation.

Data relevant to each objective, such as the role of PCT in predicting mortality and its diagnostic value in postoperative and hospital-acquired infections, were mapped and categorised. Studies employing both qualitative and quantitative methodologies were included, with each study's findings evaluated in the context of elderly surgical patients. Extracted findings were tabulated to facilitate narrative synthesis and meta-analytic procedures. This systematic review adhered to PRISMA guidelines and included 11 studies evaluating the prognostic and diagnostic value of serum PCT in elderly postoperative patients. Studies were selected based on their assessment of PCT in relation to 30-day mortality or infectious outcomes and included diverse surgical populations (orthopaedic, cardiac, thoracic, abdominal, and general procedures).

For the PCT kinetics analysis, studies reporting serial measurements of serum PCT at defined postoperative intervals (typically days 1, 3, 5, and 7-8) were extracted and compared. Mean or median PCT values were compiled from infected and non-infected cohorts. Patients were stratified by infection status and survival outcomes, and pooled estimates were used to assess temporal trends. For studies reporting comparable time points (days 5-7), we calculated pooled mean PCT levels and performed a two-tailed t-test to estimate the mean difference between the infected and non-infected groups, with significance set at p < 0.05. This descriptive statistical approach enabled visualisation of peak PCT patterns, delayed clearance, and potential prognostic thresholds indicative of adverse outcomes.

Results

**Figure 1:**
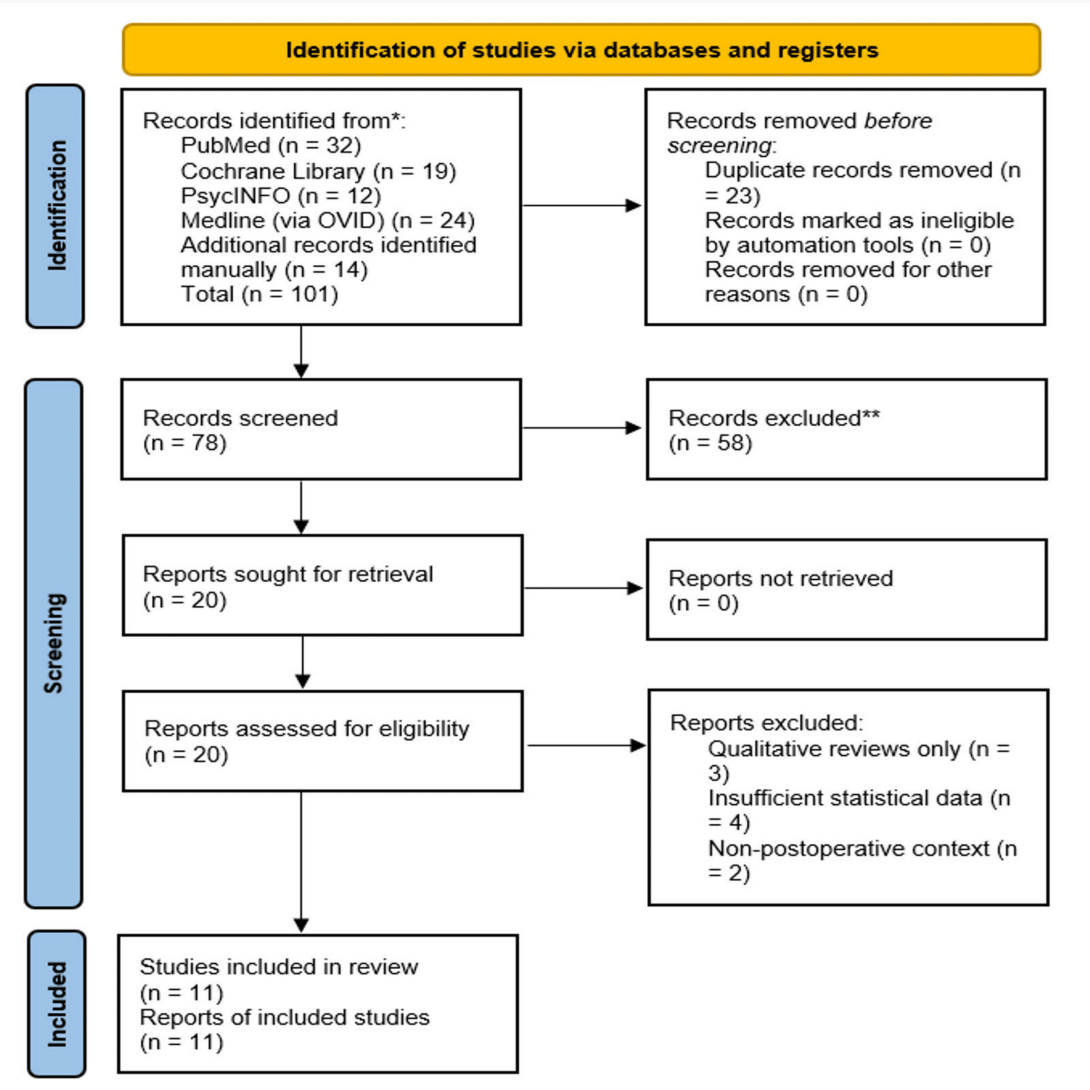
Preferred Reporting Items for Systematic reviews and Meta-Analyses (PRISMA) sheet for the final studies identified for review

The following PRISMA 2020 flow diagram (Figure 1) outlines the study identification and screening process used in this systematic review. It summarises the number of records retrieved across major databases, the exclusions applied during title/abstract screening and full-text assessment, and the final selection of studies included for synthesis. This transparent reporting framework ensures reproducibility and clarifies how the final pool of 11 studies was derived.

**Figure 2:**
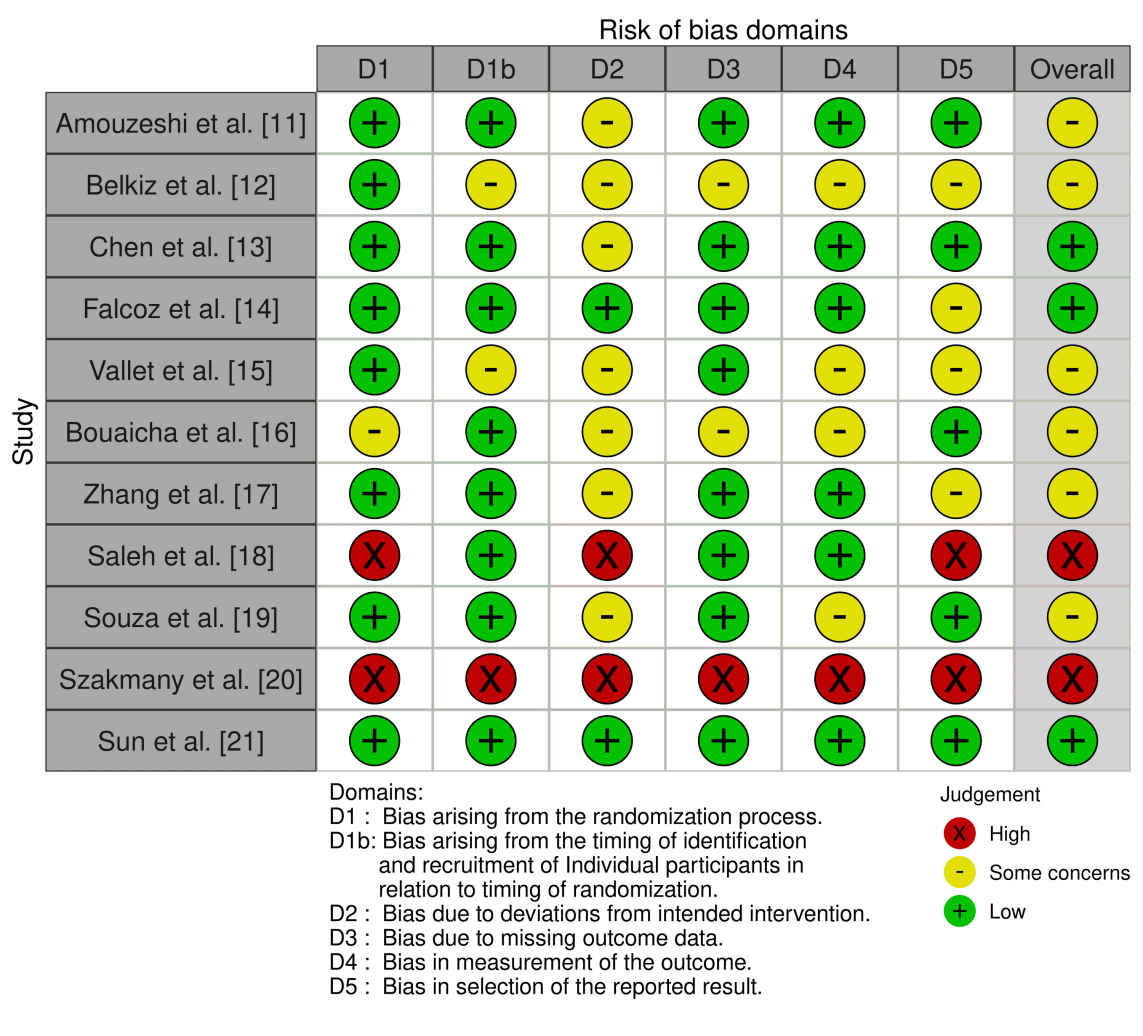
Cochrane Risk of Bias (RoB) 2 assessment across included studies

Critical appraisal was conducted using the Cochrane Risk of Bias 2 (RoB 2) tool [9], as demonstrated in Figure 2, which is designed specifically for evaluating randomised trials. RoB 2 provides a transparent and systematic approach to determine the internal validity of studies, thereby enhancing the reliability of evidence synthesis in clinical research [10]. While this process supports robust conclusions, studies with methodological limitations or substantial bias across domains may still risk misinforming clinical practice if not carefully interpreted.

Table 2 summarises the methodological features, populations, and key findings of the 11 included studies on PCT in elderly postoperative patients. By categorising data by study design, biomarker timing, and outcome relevance, such as mortality, surgical complications, and hospital-acquired infections, it highlights both the strengths and variability in the evidence. Most studies used serial PCT measurements, consistently linking elevated levels with infection, while mortality prediction remained inconsistent.

**Table 2:** Overview of the included studies on procalcitonin in elderly surgery PCT, procalcitonin; CRP, C-reactive protein; ESR, erythrocyte sedimentation rate; IL-6, interleukin-6; WBC, white blood cell count; THR, total hip replacement; CABG, coronary artery bypass grafting; MODS, multiple organ dysfunction score; AUC, area under the curve; SSI, surgical site infection; UTI, urinary tract infection; AKI, acute kidney injury; ICU, intensive care unit; SIRS, systemic inflammatory response syndrome; PJI, periprosthetic joint infection; hsCRP, high-sensitivity C-reactive protein

Author	Nature of study	Population and setting	Biomarkers used	Key biomarker insight	30-day mortality	Hospital-acquired infection	Surgical complications
Amouzeshi et al. [11] (2021)	Prospective cohort	100 post-CABG elderly patients (Iran)	PCT, CRP	PCT > 0.8 ng/ml linked to mortality and infection	Significant	Not assessed	Wound infection observed
Belkiz et al. [12] (2020)	Retrospective cohort	101 post-op geriatric patients (Turkey)	PCT, CRP, ESR	PCT not predictive of mortality; CRP/ESR better indicators	No correlation	Grouped outpatient date	Not explored
Chen et al. [13] (2023)	Nested case-control	498 elderly non-cardiac surgery (China)	PCT, CRP, hsCRP	PCT-24 hr and PCT-change predictive of complications	Limited deaths	Pneumonia, UTI	Major complications tracked
Falcoz et al. [14] (2005)	Prospective clinical	157 thoracic surgery patients (France)	PCT, CRP	PCT is superior in diagnosing hospital-acquired pneumonia	Low incidence	Confirmed pneumonia	Pulmonary complication
Vallet et al. [15] (2016)	Prospective observational	436 elderly hip fracture patients (France)	PCT, CRP, albumin, creatinine	PCT strongest mortality predictor (AUC 0.74)	Significant	Mixed infection data	General acute complications
Bouaicha et al. [16](2013)	Prospective observational	31 THR patients without infection (Switzerland)	PCT, CRP, IL-6, WBC	Stable PCT kinetics post-op defined the non-infectious baseline	Not reported	Not present	Uneventful surgeries
Zhang et al. [17] (2018)	Retrospective cohort	50 matched arthroplasty patients (China)	PCT, WBC	PCT AUC = 0.978 for diagnosing peri-op infections	Not reported	Pneumonia, UTI, SSI	Superficial infections
Saleh et al. [18] (2017)	Prospective observational	60 hip fracture elderly patients (Egypt)	PCT, CRP, WBC	PCT perfect diagnostic accuracy (Day 3 AUC = 1.000)	Not specified	Wound infection confirmed	Culture-confirmed SSI
Souza et al. [19] (2024)	Prospective observational	40 open fracture patients (India)	PCT	Elevated pre/post-op PCT linked to poor wound outcomes	Not reported	Nosocomial wound infection	Orthopaedic wound infection
Szakmany et al. [20](2003)	Prospective observational	153 major abdominal surgery patients (Hungary)	PCT	Elevated PCT in non-survivors, but inferior to the MODS score	Moderate mortality	Correlated but not isolated	MODS-related complications
Sun et al. [21] (2024)	Meta-analysis	621 patients from 9 PJI studies (Global)	PCT (various platforms)	High specificity for PJI diagnosis, poor sensitivity overall	Not mortality-focused	Focus on PJI	Prosthetic joint infections

Outcomes

30-Day Postoperative Mortality

Six studies explored the association between PCT levels and 30-day postoperative mortality in elderly patients, yielding mixed results. Vallet et al. [15] identified PCT as the most predictive biomarker (area under the curve (AUC) = 0.74) for mortality after hip fracture surgery. Amouzeshi et al. [11] found that PCT >0.8 ng/mL significantly correlated with early mortality following Coronary artery bypass grafting (CABG) (p = 0.003). Chen et al. [13] emphasised PCT kinetics over static values as predictors of complications, though direct mortality data were limited due to low event rates.

Other studies offered more cautious interpretations. Szakmany et al. [20] reported elevated PCT in non-survivors after abdominal surgery but found the multiple organ dysfunction score (MODS) to be superior in mortality prediction. Belkiz et al. [12] observed no significant difference in PCT between survivors and non-survivors (p = 0.098). Bouaicha et al. [16] documented stable PCT trends in uncomplicated Total hip replacement (THR), suggesting that deviations may be noteworthy, although mortality was not directly assessed.

Overall, while PCT alone may lack consistent predictive power for mortality, its dynamic pattern, especially sustained elevations, shows promise when interpreted alongside clinical scores. These findings support the use of serial PCT measurements as part of a broader postoperative surveillance strategy in elderly populations.

Surgical Complications (e.g., PJI and Wound Infections)

Eight studies assessed PCT as a diagnostic marker for surgical infections in elderly patients. Saleh et al. [18] reported perfect accuracy on day 3 for superficial wound infections (AUC = 1.000), while Zhang et al. [17] found high performance in detecting pneumonia and SSI (AUC = 0.978). Souza et al. [19] identified elevated PCT (>0.5 ng/mL) as a reliable infection indicator in open fractures, and Falcoz et al. [14] showed PCT outperformed C-reactive protein (CRP) for diagnosing pneumonia post-thoracic surgery (AUC = 0.92). Sun et al. [21] identified the pooled sensitivity of serum PCT for PJI diagnosis to be 0.441 (95% CI: 0.384-0.500), and the pooled specificity was 0.852 (95% CI: 0.811-0.888).

Complementary findings came from studies such as Bouaicha et al. [16], who described stable post-op PCT levels in uncomplicated THR as a potential non-infectious baseline, and Chen et al. [13], who associated elevated 24-hour PCT with various complications (AUC = 0.750). Vallet et al. [15] reported elevated PCT in patients with intensive care unit (ICU) needs or heart failure, but did not isolate infection-specific outcomes. 

Overall, PCT shows strong diagnostic specificity for localised infections and surgical wound complications in elderly patients, particularly when tracked serially between days 3-8 postoperatively. Its kinetics offer valuable early signals to support timely clinical decision-making.

Hospital-Acquired Infections

Five studies directly assessed PCT’s role in detecting hospital-acquired infections among elderly surgical patients. Falcoz et al. [14] and Zhang et al. [17] confirmed PCT’s high accuracy in diagnosing pneumonia and UTI, outperforming CRP. Chen et al. [13] linked early PCT and CRP trends with infection and ICU stay. Souza et al. [19] identified persistent PCT elevation in infected trauma cases, while Saleh et al. [18] showed that PCT could flag infections up to 48 hours before culture confirmation.

Together, these findings support PCT as a sensitive and timely biomarker for nosocomial infections, aiding early intervention and monitoring in high-risk elderly populations.

Clinical Decision-Making in Elderly Surgical Patients

All studies emphasised that PCT should be interpreted in conjunction with the clinical context rather than in isolation. Vallet et al. [15] proposed using PCT thresholds (>0.39 µg/L) to aid risk stratification and ICU referral in elderly patients. Chen et al. [13] supported serial PCT monitoring to guide perioperative triage decisions, while Saleh et al. [18] highlighted its usefulness in antibiotic management and tracking treatment response.

In addition, Falcoz et al. [14] and Souza et al. [19] advocated integrating PCT with systemic inflammatory criteria (e.g., SIRS) to support precision-targeted care. Overall, dynamic PCT measurements offer clinical value for early intervention, discharge planning, and antibiotic stewardship in elderly postoperative settings.

PCT Kinetics

To assess PCT's kinetic profile post-surgery, we extracted data from six studies reporting its serial measurements (Table 3).

**Table 3:** Average procalcitonin (PCT) values over time (ng/mL)

Study	Group	Day 1	Day 3	Day 5	Days 7-8
Bouaicha et al. [16]	Non-infected	0.21	0.28	0.15	<0.1
Saleh et al. [18]	Infected	0.3	0.30	0.35	0.33
	Non-infected	0.16	0.16	0.15	0.14
Zhang et al. [17]	Infected	-	-	0.49	0.69
	Non-infected	-	-	0.05	0.03
Souza et al. [19]	Infected	1.03	-	1.16	-
	Non-infected	<0.5	-	<0.5	-
Chen et al. [13]	Major complication	-	0.54	-	-
	No complication	-	0.35	-	-

Discussion

Summary of Evidence

This systematic review synthesised findings from 11 studies investigating the prognostic utility of serum PCT in elderly patients undergoing various surgical procedures. Across clinical contexts, serial PCT measurements offered superior insight compared to isolated baseline values, especially when interpreted alongside scoring systems and inflammatory markers.

Prognostic Value for Postoperative Mortality

PCT’s role in predicting 30-day postoperative mortality was mixed. Vallet et al. [15] and Amouzeshi et al. [11] found statistically significant associations between elevated PCT (≥0.8 ng/mL) and early mortality. By contrast, Belkiz et al. [12] and Szakmany et al. [20] reported limited prognostic accuracy. These inconsistencies likely reflect heterogeneity in surgical types, comorbidities, and biomarker timing. Importantly, studies showed improved mortality stratification when PCT was paired with scoring tools such as MODS or SIRS.

Diagnostic Utility for Postoperative Infections

PCT consistently demonstrated greater diagnostic value in detecting surgical site and hospital-acquired infections. Studies in orthopaedic and cardiothoracic settings [14,17,18,19] showed elevated postoperative PCT levels (≥0.5 ng/mL) strongly correlated with confirmed infections, outperforming CRP and WBC in sensitivity and specificity (80-100%). These associations were most robust when PCT was monitored serially.

Temporal Dynamics and Kinetic Profiles

**Figure 3:**
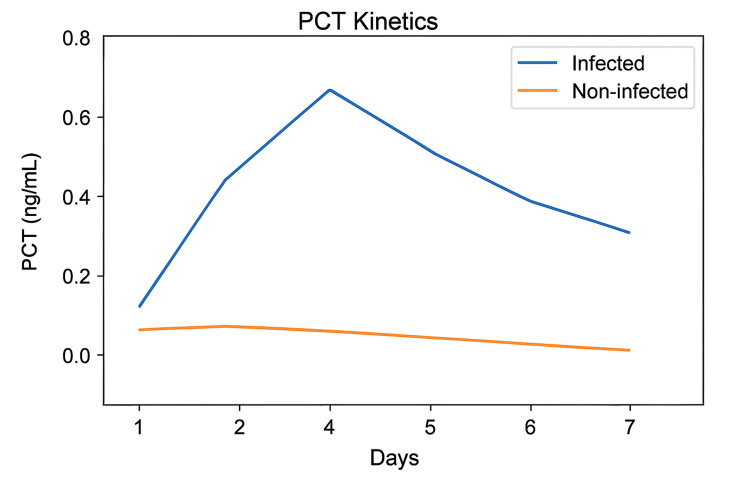
Comparison of average procalcitonin (PCT) kinetics in infected vs. non-infected elderly postoperative patients

Kinetic analysis revealed that infected patients showed peak PCT levels between days 2 and 5, with slow decline thereafter, while non-infected patients peaked mildly near day 2 (≤0.3 ng/mL) and returned to baseline. Chen et al. [13] and Amouzeshi et al. [11] linked delayed PCT decline to poor outcomes. Pooled data [17-19] in Figure 3 showed a significant mean difference of 0.55 ng/mL between infected and non-infected groups on days 5-7 (p < 0.001), confirming the clinical relevance of sustained elevation beyond 72 hours.

Implications for Postoperative Surveillance

These findings support using serial PCT measurements as part of multimodal postoperative surveillance in elderly patients. A persistent rise beyond day 3 may justify closer evaluation, prolonged monitoring, or empirical antibiotic initiation. For mortality prediction, PCT should not be used in isolation but may enhance accuracy when combined with other markers such as IL-6 or CRP and validated scoring systems.

Comparative Biomarker Performance

Although Juneja et al. [22] and Nakamura et al. [23] demonstrated that presepsin and PCT offer comparable diagnostic value for sepsis in critically ill patients, their studies were not conducted in postoperative cohorts. Notably, Nakamura et al. [23] found PCT to be significantly more accurate than presepsin in patients with severe acute kidney injury, while Juneja et al. [22] reported similar efficacy between the two biomarkers but limited predictive value for ICU mortality. These insights suggest potential utility in postoperative settings, but further large-scale trials are needed to evaluate the comparative performance of presepsin, IL-6, and PCT in predicting postoperative mortality.

## Conclusions

Serial monitoring of PCT offers meaningful value in identifying postoperative infectious complications in elderly surgical populations. Although its standalone prognostic value for mortality is limited, when interpreted dynamically and contextually, PCT contributes to improved risk stratification, early intervention planning, and postoperative care optimisation. Further multicenter trials with standardised protocols are warranted to confirm these findings and refine their clinical application.
